# Energetic and Geometric Characteristics of the Substituents: Part 2: The Case of NO_2_, Cl, and NH_2_ Groups in Their Mono-Substituted Derivatives of Simple Nitrogen Heterocycles

**DOI:** 10.3390/molecules26216543

**Published:** 2021-10-29

**Authors:** Paweł A. Wieczorkiewicz, Halina Szatylowicz, Tadeusz M. Krygowski

**Affiliations:** 1 Faculty of Chemistry, Warsaw University of Technology, Noakowskiego 3, 00-664 Warsaw, Poland; halina@ch.pw.edu.pl; 2 Department of Chemistry, University of Warsaw, Pasteura 1, 02-093 Warsaw, Poland

**Keywords:** substituent effect, heterocyclic compounds, pyridine, substituent energy

## Abstract

Variously substituted N-heterocyclic compounds are widespread across bio- and medicinal chemistry. The work aims to computationally evaluate the influence of the type of N-heterocyclic compound and the substitution position on the properties of three model substituents: NO_2_, Cl, and NH_2_. For this reason, the energetic descriptor of global substituent effect (*E*_rel_), geometry of substituents, and electronic descriptors (cSAR, pEDA, sEDA) are considered, and interdependences between these characteristics are discussed. Furthermore, the existence of an endocyclic N atom may induce proximity effects specific for a given substituent. Therefore, various quantum chemistry methods are used to assess them: the quantum theory of atoms in molecules (QTAIM), analysis of non-covalent interactions using reduced density gradient (RDG) function, and electrostatic potential maps (ESP). The study shows that the energetic effect associated with the substitution is highly dependent on the number and position of N atoms in the heterocyclic ring. Moreover, this effect due to interaction with more than one endo N atom (e.g., in pyrimidines) can be assessed with reasonable accuracy by adding the effects calculated for interactions with one endo N atom in substituted pyridines. Finally, all possible cases of proximity interactions for the NO_2_, Cl, and NH_2_ groups are thoroughly discussed.

## 1. Introduction

Classically, the problem of substituent effects is mainly related to approaches that analyze their influence on the physical or chemical properties of the substituted systems. However, the substituent itself, when interacting with substituted moieties, not only affects the substituted molecule but can also undergo structural changes. This paper targets the problem of changes in the structure and energy of the substituent caused by its interaction with the substituted system. To assess these changes for substituents of various nature, we chose strongly electron-attracting and electron-donating groups, NO_2_ and NH_2_, respectively, and, additionally, Cl, as a substituent for some neutral properties of this nature. These groups, attached at various positions of simple N-heterocyclic systems, allow presenting their behavior both in the vicinity of the endocyclic nitrogen atom(s) and in cases where such interactions do not occur, but electronic interactions should be formally comparable. Thus, three different cases should be considered:

- when the group is neighboring two endocyclic nitrogen atoms;
- when only one nitrogen atom is in *ortho* position to the group; and
- when neighbors are only CH groups.

**Figure 1:**
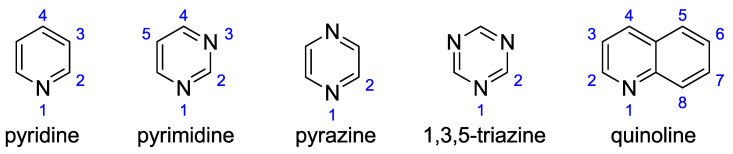
Studied N-heterocyclic compounds with the atom numbering scheme.

In all these cases, apart from group energy differences, the substituents may be subject to intramolecular interactions, resulting in changes in bond lengths and valence angles. In this study, the variability of substituent parameters under the influence of proximity interactions is the subject of more detailed analyzes. The studied N-heterocyclic molecules are shown in Figure 1; each of the selected groups was attached to a carbon atom in a position marked with a blue number.

Six-membered nitrogen heterocycles are an important subject of research due to their importance in organic, bioorganic, and medicinal chemistry [1,2]. They are widely used as pharmaceuticals, agrochemicals, and natural products [3,4,5]. In addition to their use as ligands for complexing metals [6], they are valuable in the target-oriented synthesis of biologically active molecules [7,8,9,10] and new materials [11]. Undoubtedly, one of the most important pyrimidine derivatives are the nucleic acid bases: cytosine, thymine, and uracil, while adenine and guanine contain a pyrimidine ring [12,13].

The substituents in mono-substituted polycyclic aromatic hydrocarbons can only interact with adjacent hydrogen atoms. For the nitro and amino groups, it has been shown that their energies strongly depend on the effects of proximity [14,15]. Moreover, it was found that, in these systems, the energetic and geometric properties of the substituents are highly interdependent. In the case of mono-substituted six-membered N-heterocyclic molecules (Figure 1), the variation in the proximity effects is significantly greater. Thus, a question arises about the influence of the endocyclic nitrogen atom(s) on the substituent’s structural, electronic, and energetic parameters.

## 2. Methodology

B3LYP functional [16] with 6-311++G(d,p) [17] basis set was chosen as a computational method. Calculations were performed in Gaussian 16, Revision C.01 program [18]. For all systems, frequency calculations were performed after geometry optimization. No imaginary frequencies were found.

**Scheme 1:**
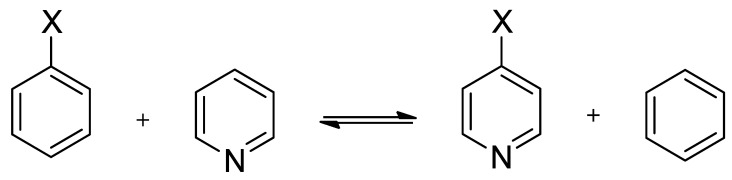
Homodesmotic reaction used in the calculation of *E*_rel_(X) in 4-X- substituted pyridine (X = NO_2_, Cl, NH_2_).

Substituents were described in terms of the relative energy of the substituent, *E*_rel_(X) [14]. It is defined as an energetic effect of the homodesmotic reaction shown in Scheme 1. Benzene was used as a reference system for all studied heterocycles.

*E*_rel_(X) was calculated from Equation (1):

(1) $$E_{\mathrm{rel}}\left(X\right)=E\left(\mathrm{RX}\right)+E\left(\mathrm{benz}\right)-E\left(\mathrm{benzX}\right)-E\left(R\right),$$

where *E*(RX), *E*(benz), *E*(benzX), and *E*(R) are the electronic energies of the substituted R-X system, benzene, X substituted benzene, and unsubstituted R heterocycle, respectively. Hereinafter, *E*_rel_(X) is abbreviated as *E*_rel_. This parameter evaluates the energetic effect of interactions between the substituent and the substituted system. Negative values correspond to stabilization of the molecule with respect to the reference system, whereas the positive values indicate destabilizing interactions.

The cSAR (charge of the substituent active region) index [19,20] was used to characterize the electron-accepting/donating properties (EA/ED) of the substituents. For the X substituent, it was calculated according to Equation (2):

(2) $$\mathrm{cSAR}\left(X\right)=q\left(X\right)+q(C_{\mathrm{ipso}}),$$

where *q*(X) is a sum of charges of atoms at substituent X, and *q*(C_ipso_) is the atomic charge at ipso carbon atom. Atomic charges were evaluated using the Hirshfeld method [21]. The interpretation of the cSAR(X) values is as follows: the electron-donating strength of the X group increases with the increase of cSAR(X), whereas the electron-accepting strength decreases.

Other descriptors of EA/ED properties of substituents, pEDA(X), and sEDA(X) [22] (pi and sigma Electron Donor-Acceptor index, respectively), were calculated using Equations (3) and (4):

(3) $$\mathrm{pEDA}\left(X\right)=\overset{n}{\underset{i=1}{\sum }}\pi_{X}^{i},$$

(4) $$\mathrm{sEDA}\left(X\right)=\overset{n}{\underset{i=1}{\sum }}\sigma_{X}^{i},$$

where *n* is a number of atoms in the substituent X, and $\pi_{X}^{i}$ is the occupancy of 2pz natural atomic orbital (NAO) of a given atom of substituent (assuming that rings lie in the XY plane), whereas $\sigma_{X}^{i}$ is the sum of occupancies of 2s, 2py, 2px NAOs of this atom. They are often used to evaluate resonance (pEDA(X)) and inductive (sEDA(X)) substituent effects [23,24]. The values of pEDA and sEDA are often compared to some reference system. The differences are interpreted as an estimation of the π and σ electron flow as a result of changing the substituent (X) or the substituted system (R). In addition, they can be used to characterize both the substituent and the substituted moiety.

∆pEDA(X) and ∆sEDA(X) indicate changes in the pEDA(X) and sEDA(X) values of substituent X with respect to their values in substituted benzene (in the case of six-membered rings: pyridine, pyrimidine, pyrazine, triazine) or 2-X-naphthalene (for quinoline).

It should be stressed, that *E*_rel_ is a global descriptor of the substituent effect, i.e., it is calculated for the entire molecule. The cSAR and EDA descriptors are the local descriptors, meaning they are calculated only for the substituent or other fragment of the substituted system. For this reason, the latter can be compared with the appropriate parent system (e.g., nitroquinolines with 1- or 2-nitronaphtalene, aminopyridines with aniline).

Natural population analysis was performed using NBO 6.0 software (Theoretical Chemistry Institute, University of Wisconsin, Madison, WI, USA) [25]. Quantum theory of atoms in molecules (QTAIM) calculations and analyses were performed using AIMAII software [26].

For analysis of non-covalent interactions, a reduced density gradient (RDG) function was used [27]. This function is a modification of electron density (*ρ*) and its gradient (∇*ρ*) (Equation (5)).

(5) $$\mathrm{RDG}=\frac{1}{{\left[2\cdot {\left(3\pi \right)}^{2}\right]}^{1/3}}\cdot \frac{\left|\nabla \rho \left(r\right)\right|}{\rho {\left(r\right)}^{4/3}}.$$

Analysis of RDG isosurfaces is frequently used [28,29] to visualize the non-covalent interactions between groups of atoms. Additionally, the character of these interactions can be assessed by looking at the value of sign(λ_2_)∙*ρ* (where λ_2_ is the second eigenvalue of the Hessian matrix of *ρ*) at each point of the isosurface. Negative values indicate strong non-covalent interactions, such as the hydrogen bonding, around zero weak ones (van der Waals), whereas positive are non-bonding ones, e.g., the steric repulsion. Algorithms for the calculation of RDG implemented in Multiwfn software were used [30]. Electrostatic potential (ESP) [31] maps were generated in the Multiwfn program [32]. For visualization of ESP maps and RDG isosurfaces, the VMD program was used [33].

## 3. Results and Discussion

The discussion of the results is presented in three parts. The first is devoted to the energetic characteristics of the substituents. Then, changes in the electronic and structural properties of the substituents are discussed. The last part is about the proximity effect. The obtained values of the considered descriptors of the substituent effect for all studied systems are presented in Tables S1 and S2 (Supplementary Materials).

### 3.1. Energetic Characteristics of Substituents

The energy of the substituent, *E*_rel_, can be calculated concerning various reference systems. However, by using benzene as a reference system (Scheme 1), it is possible to differentiate signs between the electron-withdrawing group, NO_2_ (+), and electron-donating group, NH_2_ (−), as shown in Table 1. In the case of X = NH_2_, the *E*_rel_ values range from 1.01 to −15.8 kcal/mol. 5-X-quinoline is the only case where *E*_rel_ is substantially positive. On the other hand, the nitro substitution of the studied heterocycles results in the positive energy effect only, *E*_rel_, ranging from 0.59 to 8.55 kcal/mol. Chlorine substitution leads to *E*_rel_ values varying from −2.25 to 2.60 kcal/mol. So, contrary to the strongly electron-donating or withdrawing amino and nitro groups, the values are positive or negative. It can be concluded that swapping one or more carbon atoms for nitrogen atoms in the aromatic ring generally results in a decrease in the stability of the nitro-substituted systems and an increase in the stability of the amino-substituted compounds. Therefore, regarding X = NO_2_ and NH_2_, electronic interactions between the substituent and the substituted system are a major factor affecting the stability of the substituted molecule. Other effects, such as the substitution position, especially the *ortho* effect, can change the extent of the stabilization but cannot change the character (stabilizing/destabilizing) of the interaction between the X group and the substituted N-heterocyclic moiety.

Interestingly, the *E*_rel_ values derived from additivity of interactions (*E*_rel-add_) in some cases differ only slightly from the values obtained from Eq. (1). In order to calculate *E*_rel-add_, three cases of intramolecular interactions with endocyclic nitrogen atom were considered: (i) *ortho (o)*, as in 2-X-pyridine, (ii) *meta (m)*, as in 3-X-pyridine, and (iii) *para (p)*, as in 4-X-pyridine. For example, the *E*_rel-add_ for 4-NO_2_-pyrimidine (*o*, *p*) was calculated as the sum of the *E*_rel_ in the *ortho* (2-X-pyridine, 2.27 kcal/mol) and *para* (4-X-pyridine, 3.01 kcal/mol) systems: *E*_rel-add (o,p)_ = *E*_rel(o)_ + *E*_rel(p)_. Hence, more complicated intramolecular interactions can be approximately separated into a sum of simpler ones. For 5-, 6-, and 7-X- quinolines, the *E*_rel_ values differ only slightly from the *E*_rel_ values for 1- and 2-X- naphthalenes (*E*_rel-add_ given in Table 1) [14]. This indicates that the N atom in the adjacent ring does not strongly affect the substituent energy.

The systems in which *E*_rel-add_ highly differs from *E*_rel_ are characterized by significant proximity interactions. For example, in 2-NO_2_-pyrimidine, the existence of two O∙∙∙N interactions causes a substantial rotation of the nitro group around the C−N bond (by 56°), which further increases the destabilization of this system. Consequently, the additivity of interactions predicts a lower than the actual *E*_rel_ value. Other systems where such rotation occurs are the 4-NO_2_-quinoline (35°), 5-NO_2_-quinoline (28°), 8-NO_2_-quinoline (58°), and NO_2_-triazine (66°). Substantial difference between *E*_rel_ and *E*_rel-add_ also occurs in 8-NH_2_-quinoline, where N∙∙∙HN H-bond is present (see Section 3.3). In the case of X = Cl, such differences can be observed for (*o*, *o*) systems (X-triazine, 2-pyrimidine).

Despite the repulsive O∙∙∙N contacts occurring in *ortho* nitro substituted heterocycles, the energetic effect of such interactions is smaller than in *meta* and *para* systems. This is clearly seen by comparing 2-, 3-, 4-NO_2_-pyridines and 2-, 3-, 4-NO_2_-quinolines. Another example of this effect can be seen in 4- and 5-NO_2_-pyrimidines. The (*m*, *m*) 5-derivative has the *E*_rel_ value higher by 0.32 kcal/mol than the (*o*, *p*) 4-system. The difference is similar to that between 2- and 3-NO_2_-pyridine (0.36 kcal/mol).

As the *E*_rel_ values show, the *para* interaction between the nitro group and the endocyclic N atom leads to the highest destabilizing effect in the considered heterocycles.

Substitution by the chlorine results in values of *E*_rel_ that are both positive and negative. The latter are observed only in the *ortho* systems. It should also be noticed that Cl derivatives are characterized by a smaller range of *E*_rel_ variability than NH_2_ and NO_2_.

**Scheme 2:**
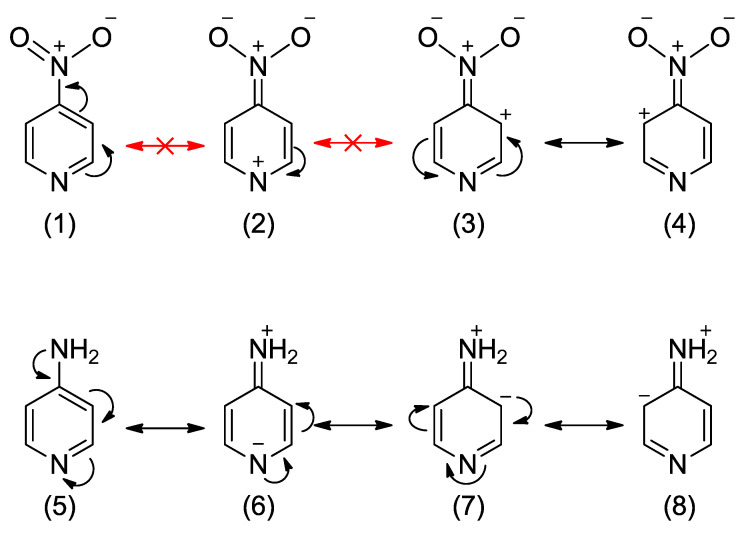
Resonance structures of 4-NO_2_-pyridine and 4-NH_2_-pyridine.

Regarding the substitution by the electron-donating amino group, the most energetically favored is the *ortho* position with respect to the endocyclic nitrogen. The exchanging of more carbons for nitrogens in the ring results in better stabilization, as shown for pyrimidines and triazine in Table 1. Interestingly, unlike in the nitro derivatives, the *meta* interaction does not lead to any energetic effects (*E*_rel_ ~ 0 kcal/mol). It means that the *meta* interaction with endo nitrogen (or double *meta*, as in 5-NH_2_-pyrimidine) is energetically the same as if the ring consisted of only carbon atoms (benzene). The *E*_rel_ values for 4-NO_2_- and 4-NH_2_- pyridines indicate that *para* interactions of nitro and amino groups have roughly the same absolute energetic effect (~3 kcal/mol). However, the direction of effects is opposite—stabilization in amino and destabilization for nitro derivatives. A simple explanation of this phenomenon comes from the analysis of resonance structures of 4-NO_2_ and 4-NH_2_-pyridine, shown in Scheme 2. In a very unlikely structure (2), with a positive charge at the highly electronegative nitrogen atom, the resonance effect of the NO_2_ group has to be significantly disturbed. On the other hand, in the NH_2_ derivative, the structure (6) has a negative charge on the nitrogen atom, which is more likely than a negative charge on the less electronegative carbon atom (as in aniline). Hence, the resonance effect of the NH_2_ group is likely to be strengthened compared to that in aniline. Both the disruption of resonance for the NO_2_ and the strengthening for the NH_2_ are reflected in the electron-donating/withdrawing properties of substituents (see below).

### 3.2. Electronic and Geometric Properties of Substituents

Electronic properties of substituents, described by the cSAR index, provide further information on the reverse substituent effect. Data gathered in Table 2 show that electronic properties are highly dependent on the position of substitution. The electron-donating/accepting strength of considered substituents in heterocycles can be compared with the appropriate strength in nitro-(cSAR(X) = −0.138), amino-(cSAR(X) = +0.094), and chlorobenzene (cSAR(X) = −0.051). All but one substitution of heterocycles with the NO_2_ group result in lower EA strength of the NO_2_ than in nitrobenzene. In 3-NO_2_-quinoline, its slightly higher EA ability may come from the existence of a second aromatic ring, which can lend its π-electrons to the NO_2_ group. In this case (and other quinolines), it is more reasonable to compare cSAR(X) to the appropriate value in 2-nitronaphthalene (−0.148) [14]. Thus, replacing carbon with nitrogen highly influences the electronic interactions of NO_2_ with the aromatic system, lowering the EA strength of this group. This is also reflected in the ∆pEDA values, which indicate a lower (relative to all-carbon substituted system—X-benzene or 2-X-naphthalene) occupancy of 2p_z_ natural atomic orbitals of atoms in the substituent (i.e., less electrons are transferred from the substituted moiety to the NO_2_ group). On the other hand, all NH_2_ substituted heterocycles have a higher ED strength as compared to aminobenzene, so the opposite effect to the nitro substitution is observed. This is clearly seen from all-negative ∆pEDA(X) values (Table 2) (i.e., more electrons are transferred from the NH_2_ group to the substituted moiety). This further supports the interpretation of heteroatom effect given in Scheme 2.

**Figure 2:**
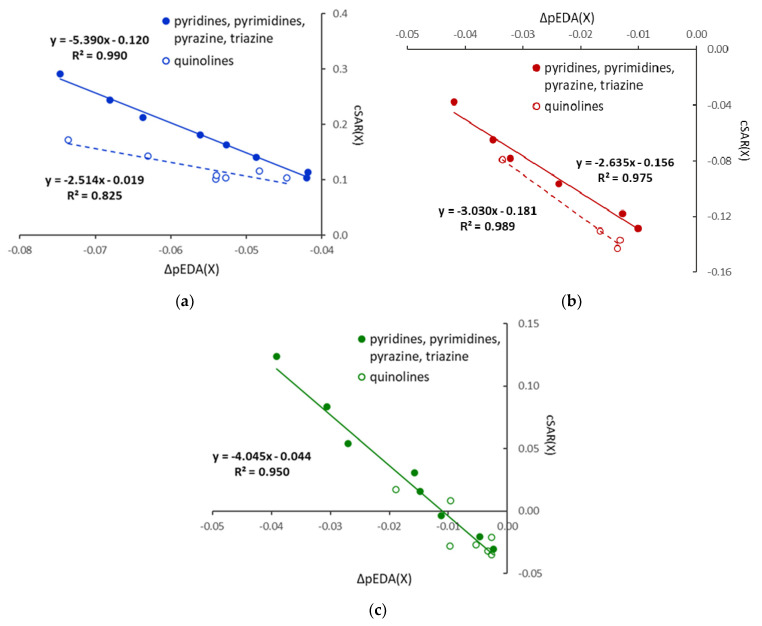
Relationships between electronic characteristics of the substituents, cSAR(X) and ∆pEDA(X) for X = NH_2_ (**a**), NO_2_ (**b**), and Cl (**c**); for nitro derivatives, only planar systems are considered.

**Figure 3:**
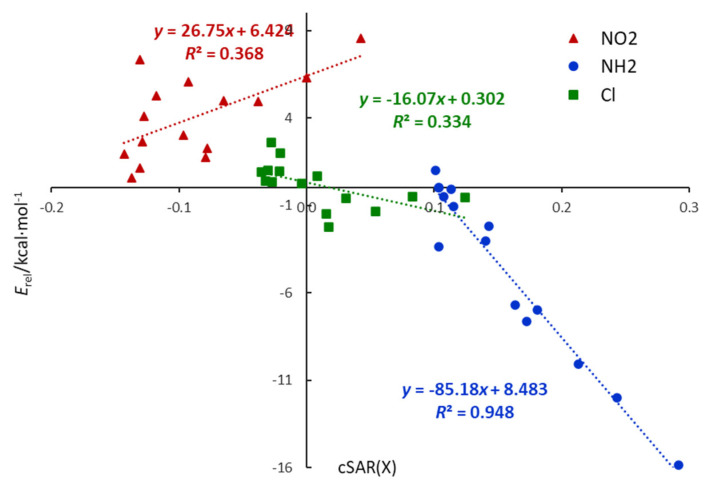
Relationships between electronic, cSAR(X), and energetic, *E*_rel_, characteristics of the substituent in monosubstituted N-heterocycles.

Substitution by Cl leads to cSAR(X) values higher than that in chlorobenzene, even positive for *ortho* derivatives. Thus, the electronic properties of Cl are slightly shifted towards electron donation. Additionally, relations in Figure 2 show that cSAR(X) and ∆pEDA(X) electronic parameters are well correlated, with a clear separation of six-membered heterocycles and quinolines; however, for the nitro derivatives, only planar systems are considered. The cSAR(X) parameter does not correlate well with the *E*_rel_ energetic parameter for X = Cl and NO_2_; however, it correlates well (*R*^2^ = 0.948) for the X = NH_2_ series (Figure 3). It shows that changes in the electronic structure of the substituent are only partly responsible for *E*_rel_ variability. In other words, cSAR is the local substituent effect parameter, while *E*_rel_ characterizes the global substituent effect, including proximity interactions. In the case of X = NH_2_, *ortho* NH∙∙∙N interactions are responsible for a high stability increase, which is accompanied by an increase in the ED power of the substituent due to favorable electronic interactions (Scheme 2). Thus, for the NH_2_ series, these two parameters are well-correlated.

Susceptibility of ED/EA properties of X groups to the reverse substituent effect can be illustrated by ranges of variation of the cSAR(X) index. Thus, in terms of electronic properties, the NH_2_ group is the most sensitive to changes in the substituted moiety, the NO_2_ group is slightly less, and the Cl substituent is the least susceptible (the ranges of their cSAR variation are 0.190, 0.186, and 0.159, respectively).

Considering the particular substitution positions for the NO_2_ group, the substitution in the *ortho* position to the endocyclic N atom gives the lowest absolute values of the cSAR index. In the case of such “double *ortho*” substitution, in 2-NO_2_-pyrimidine and NO_2_-triazine, the cSAR values are even slightly positive. Apart from *ortho* interactions, the sterically induced rotation of the nitro group is another factor that weakens its electron-withdrawing power in these systems. Comparing the *meta* and *para* positions is particularly insightful, as any differences result from various electronic interactions. In the *meta* substitution, the nitro group possesses the highest EA strength. However, looking at ∆pEDA values, this should be rather attributed to a weaker disruption of the resonance effect by N-heteroatom in the *meta* position than to the enhancement of other electronic effects. As mentioned earlier, the *para* substitution is energetically unfavorable, resulting in the weakest EA properties of the NO_2_. It indicates a weakened π conjugation with the aromatic ring, also supported by lower occupancies of 2pz orbitals on the substituent, compared to the *meta* derivatives, as shown by ∆pEDA (Table 2). Furthermore, the weaker conjugation leads to an increase in CN bond lengths (Table 3), lower *ρ*_BCP_(CN) values (Table S1), and an increased *E*_rel_. Hence, the conclusion drawn from the simple analysis of the resonance structures (Scheme 2) can be confirmed by electronic descriptors obtained from the ground state wavefunction. An interesting observation can be made by looking at ∆pEDA(X) and ∆sEDA(X) values of systems where rotation of NO_2_ occurs. Rotation significantly weakens electron-withdrawing by resonance (highly negative values of ∆pEDA), but this seems to be somewhat compensated by an enhanced inductive effect (positive values of ∆sEDA).

The amino group, as already mentioned, is stabilized in N-heterocycles with respect to benzene. Exceptional stabilization occurs in the *ortho* position, where favorable proximity interactions are present. The NH_2_ group is highly electron-donating in the *ortho* position, as shown by the cSAR values in Table 2. Low values of pEDA also point out to high ED resonance effect of this group. Moreover, CN bonds are relatively short (Table 3), especially in double *ortho* derivatives where two attractive NH∙∙∙N interactions are present. Shortening of the CN bond and a significant increase in ED properties also occur in *para* derivatives. In this case, purely electronic interaction between the NH_2_ group and the *endo* N atom can be assumed. Its stabilizing and ED enhancing nature can be explained by resonance structures, shown in Scheme 2. Moreover, the most robust ED character of the amino group is observed for amino-triazine, where double *ortho* and *para* effects are present. It is documented by both the largest cSAR(X) (0.292, Table 2) and the shortest CN bond length (1.347 Å, Table 3). *Meta* interaction, which is energetically neutral, only slightly affects the electronic properties of the substituent. In 3-NH_2_-pyridine, the cSAR value is higher by about 0.01 than that of aniline, which is much less than in *ortho* (0.07) and *para* (0.05) pyridine derivatives. Similar conclusions can be drawn from analyzing amino-substituted quinolines and pyrimidines. Additionally, when comparing *meta* systems to *para* and *ortho*, the CN bond for *meta* is longer by about 0.01 Å in pyridine and quinoline, and 0.02 Å in pyrimidine. However, it is still shorter by ~0.005 Å than that in aminobenzene. The shortened CN bonds are another implication of enhanced π-resonance with respect to aminobenzene.

Chlorine differs from the NH_2_ and NO_2_ groups due to the much weaker resonance effect. It is well known, however, that it can act in two directions, i.e., withdraw electrons by inductive effect and donate by resonance. As mentioned earlier, *ortho* substitution leads to the positive values of cSAR(X), indicating ED properties, and its highest values are present in double *ortho* systems (2-Cl-pyrimidine, Cl-triazine). Interestingly, these are the only cases for X = Cl when *E*_rel_ is negative. In contrast, *meta* derivatives are characterized by the lowest values (negative), i.e., the strongest EA properties. *Para* interaction weakens the EA properties of the chlorine, as can be seen, for example, by comparing the cSAR(X) of 3-and 4-Cl-pyridine (Table 2). In *meta* and *para* cases, cSAR(X) values are positive, indicating electron donation. It follows that, in Cl derivatives, its ED properties are associated with negative *E*_rel_ (stabilization), whereas EA with positive *E*_rel_ (destabilization). It is well illustrated by the two groups of points for X = Cl laying in two quarters of the coordinate system in Figure 3.

For all groups, the substitution of various positions in the full-carbon ring of quinoline (5-, 6-, 7-, 8-) leads to cSAR(X) values that do not differ substantially.

**Figure 4:**
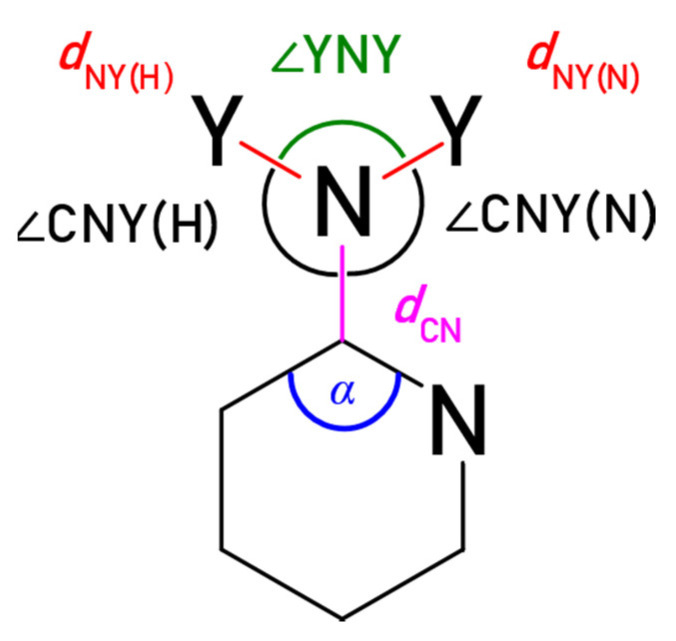
Geometric characteristics of the substituent. Y = H or O. In the case of X = Cl, only *d*_CCl_ and α can be considered. When endo N atom is not in *ortho* position, (N) and (H) denote the side closer and further (moving along the bonds) to the N atom, respectively.

The substituent X can also be described by its geometry (Figure 4). The geometry of substituent carries information about electronic interactions, as well as the proximity interactions, such as steric effects and hydrogen bonding. The obtained structural parameters are collected in Table 3, Table 4 and Table S2; the ∆α is the difference between the angle α for substituted and unsubstituted molecules.

The data summarized in Table 3 and Table 4 show that the geometry of the substituent depends not only on the substituted moiety but also influences the angle *α* (at *ipso* ring carbon atom). The ED substituent (NH_2_) reduces the *α* angle with respect to the unsubstituted system (negative ∆*α* values). The nitro group has an opposite effect, causing its increase (positive ∆*α* values). Substitution by chlorine atom leads to an increase of *α*, but a lower extent than the NO_2_ group. It is consistent with the weaker EA strength of Cl (Hammett substituent constant, *σ*_p_ = 0.23), than NO_2_ (*σ*_p_ = 0.78) [34]. Additionally, the ∆*α* ranges of variation are higher for NO_2_ (1.35°) and NH_2_ (1.12°) than for Cl (0.68°). It can also be attributed to the fact that the NO_2_ and NH_2_ groups exhibit a much higher variation in electronic properties than Cl and participate in specific *ortho* interactions. Bond length *d*_CX_ (X = N or Cl) has the highest variability for X = NH_2_ (0.0492 Å, 3.6% of the average bond length); for X = NO_2_, it is slightly lower (0.0415 Å, 2.8%), and it is the lowest for X = Cl (0.0260 Å, 1.5%). It is understandable since the Cl group does not participate in through-space proximity interactions, as NH_2_ and NO_2_, and its electronic properties are less susceptible to change due to lack of the resonance effect.

As shown in Table 3, NH_2_ substitution in the *ortho* position results in the smallest changes in α angle. *Meta* substitution results in a 23% larger change, whereas *para* substitution causes the highest decrease of α (43%). In the case of 8-NH_2_-quinoline, the most significant change in α occurs, which is probably a geometric distortion related to the formation of a favorable NH∙∙∙N hydrogen bond. This hydrogen bond fulfills the Koch-Popelier criteria [35] (*ρ*_BCP_ = 0.0170, ∇²*ρ*_BCP_ = 0.0766 a.u.). In previously investigated polycyclic aromatic hydrocarbons [14], the *E*_rel_ value obtained for the structurally similar 1-aminonaphthalene (differing only in the endo-N atom) equals 0.96 kcal/mol. Hence, the difference between *E*_rel_ for 8-NH_2_-quinoline and 1-aminonaphthalene (−4.34 kcal/mol) can be considered an approximation of the stability gain due to the existence of a hydrogen bond. The value of the ∠HNH angle is highly related to the electronic properties of the NH_2_ group (Table 5). The most important factors in this case, however, are proximity interactions. *Ortho* systems show the highest values of ∠HNH, which indicates how the NH_2_ group geometry is changed upon the formation of attractive NH∙∙∙N interactions. Generally, when strongly ED enhancing and stabilizing *ortho* and *para* electronic interactions occur, the ∠HNH angle is greater. For the substituted pyridine derivatives, it is 115.3° and 114.1°, respectively, while, for 3-NH_2_-pyridine, it is 112.5°. The highest value corresponds to a system with double *ortho* and single *para* interaction—NH_2_-triazine (120.9°). Additionally, the differences in its values between 4-NH_2_-pyrimidine (117.6°) and NH_2_-pyrazine (115.7°), or 3- and 4-NH_2_-pyridine, can be solely associated with stronger *para* interactions in 4-NH_2_ derivatives than *meta* in 3-NH_2_-pyridine and NH_2_-pyrazine.

Regarding the NO_2_ substitution, the most significant changes in the α angle occur for double-*ortho* systems. In these molecules, the NO_2_ group undergoes rotation along the CN bond to reduce steric strain. It significantly disturbs the resonance effect of the NO_2_ group, due to which the highest ∠ONO angles are observed.

As already mentioned above, the range of variability of ∆α is the smallest for the Cl substitution (Table 4). Moreover, in all cases, the ∆α values are lower than for chlorobenzene (∆α = 1.42°, Table S1). The same goes for its electron-accepting ability (in Cl-Ph cSAR(Cl) = −0.0505, Table S2). Thus, the ring nitrogen atom(s) decreases the EA strength of the Cl group.

Changes in geometry resulting from the electronic interactions with the endo-N atom can be noticed by looking at the α angles. According to the Bent-Walsh rule [36,37], α angle should be well-correlated with the electronic properties of the substituent. As shown in Figure S2, the determination coefficients (*R*^2^) of the obtained relations are less than 0.7.

However, considering only six-membered derivatives, the *R*^2^ increases and is greater than 0.75. So, the Bent-Walsh rule works. In the case of quinolines, both the position of the substitution and the additional ring influence the geometry and properties of the substituents. For example, for 4, 5, and 8 positions in quinoline (as marked on Scheme 1), a significant steric strain is imposed upon substitution, which perturbs the geometric parameters and lowers determination coefficients.

Pearson correlation coefficients (*cc*) between the pairs of the considered geometric, electronic, and energetic parameters of the six-membered heterocyclic derivatives are gathered in Table 5 and Table 6. It can be immediately noticed that, for the NH_2_ group, *cc* values are higher, and more parameters are mutually correlated. For the NO_2_ substitution, less parameters are well correlated. In this case, worse correlations may result from significant proximity interactions of the bulky NO_2_ group, that are present in *ortho* derivatives. The least significant correlations exist for Cl, where only relations between ∆α, cSAR, and pEDA have an absolute value of *cc* above 0.80. This is probably due to smaller variability of the parameters in Cl derivatives than in NH_2_ and NO_2_.

Indicative are not only the absolute values of *cc* but also their signs. The sign of *cc* is consistent with the sign of the slope in the linear equation between the parameters under consideration. For example, in the *E*_rel_ vs cSAR relation for nitro derivatives, the slope is positive (*cc* = 0.82), while negative for amine systems (*cc* = −0.99). It is illustrated, but for all tested derivatives, in Figure 2.

In the case of nitro derivatives, changes in the structural parameters are generally consistent with the Bent-Walsh rule. Increasing the α angle causes the CN bond to be lengthened (*cc* = 0.73), which results in a *d*_NY(N)_ bond shortening (*cc* = −0.95). Increasing the angle α also increases the angle ∠YNA (*cc* = 0.86). The latter is also observed for amine systems, but changes in other geometric parameters of the amine group do not comply with the Bent-Walsh rule. For example, increasing the α angle shortens the CN bond (*cc* = −0.81). However, much better correlations between pairs of its structural, electronic, and energy parameters were found.

### 3.3. Proximity Interactions

Proximity interactions are clearly noticeable when analyzing NO and NH bond lengths, as well as ∠YNY and ∠CNY angles. In nitro-substituted heterocycles with one N atom in the *ortho* position (2-X-pyridine, X-pyrazine, 2-X-quinoline, 4-X-pyrimidine) the NO bonds directed towards the *endo* N atom are shorter by about 0.014 Å than those directed towards the H atom (Table 3). Additionally, the ∠ONO angles are slightly larger, and there is a marked discrepancy between the two ∠CNO angles. In *ortho* amino-substituted heterocycles, differences in length between the two NH bonds are very slight (few thousands of Å); however, the NH(N) bonds are always longer, as a consequence of NH∙∙∙N interaction. The ∠CNH(N) angles are lower than ∠CNH(H), which is another proof of the attractive character of the NH∙∙∙N interaction.

**Figure 5:**
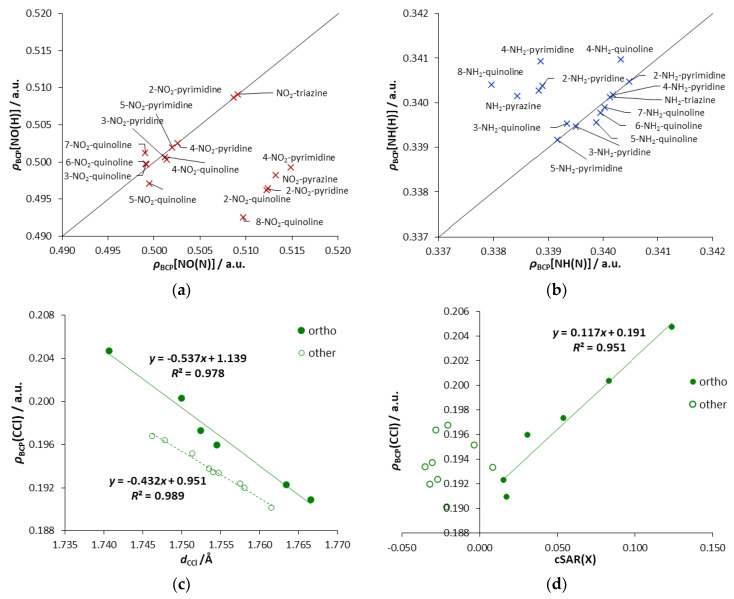
Relationships containing QTAIM parameters for nitro (**a**), amino (**b**) and chlorine (**c**,**d**) derivatives.

Asymmetricity of interactions can also be expressed with the values of *ρ*_BCP_(NY), as shown in Figure 5a,b. Moreover, the character of these interactions can also be seen in these plots. Points below the y = x line have an increased electron density at BCP[NY(N)] compared to BCP[NY(H)]. It is connected with the NO(N) bond’s shortening due to the repulsive interaction, for derivatives when only one *endo* N atom is in the *ortho* position. Points above the y = x line are slightly depleted of electron density, indicating the presence of an attractive interaction. The reduction of the electron density is related to the elongation of the NH(N) bond (Table 3), which may be due to its participation in hydrogen bonding. Such depletion is one of the criteria for the existence of hydrogen bond, as pointed out by Koch and Popelier [35].

**Figure 6:**
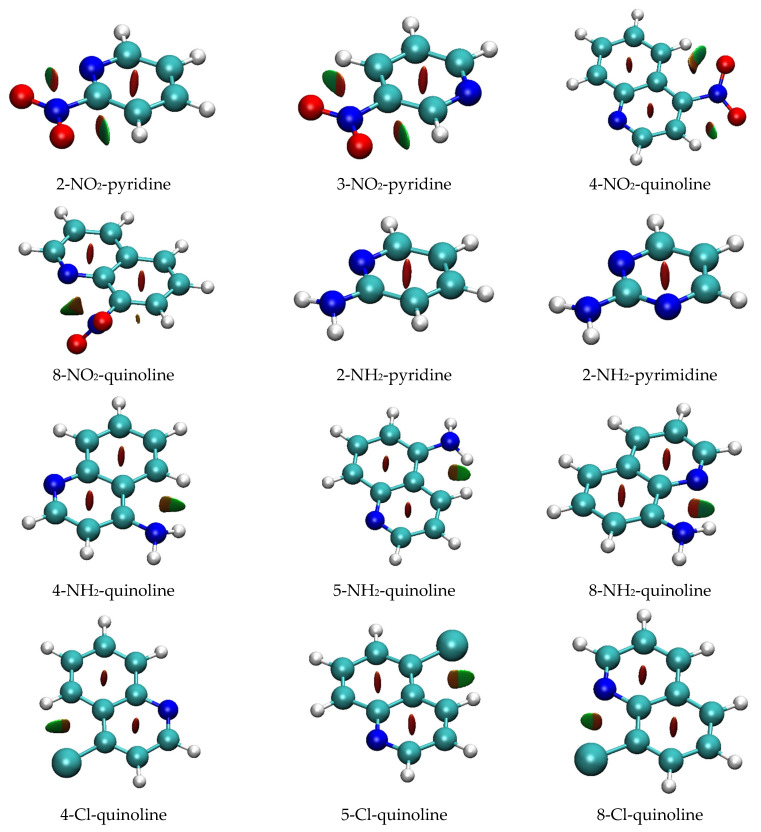
RDG isosurfaces (at RDG = 0.50). Isosurfaces are colored according to the value of sign[λ_2_(r)]**∙***ρ*(r); highly negative values are in blue (indicating strong H-bonding interaction), around zero are in green (van der Waals interaction), and highly positive are in red (steric repulsion).

However, in this case, other criteria, such as the existence of a bond critical point between the two hydrogen-bonded atoms, are not fulfilled. Moreover, the reduced density gradient (RDG) function analysis does not reveal a bonding character of NH∙∙∙N interactions (Figure 6; data for all systems are shown in Figure S3). The low NH∙∙∙N angle and the H∙∙∙N distance (in 2-NH_2_-pyridine, respectively: 71° and 2.430 Å) are possible reasons for this. The only NH∙∙∙N interaction detected by RDG analysis (at 0.50 isosurface) appears in 8-NH_2_-quinoline (H∙∙∙N distance: 2.286 Å, NH∙∙∙N angle: 104°). As mentioned earlier, for this interaction, a bond critical point was detected. Detection by both approaches indicates its superior strength compared to other proximity interactions. Close NH∙∙∙HC contacts in 4- and 5-NH_2_-quinolines (respectively, 2.056 and 2.120 Å) may indicate dihydrogen interactions. These interactions are detected by RDG as van der Waals in character (Figure 6). Moreover, in 4-NH_2_-quinoline, where closer contact exists, QTAIM analysis indicates the existence of a bond critical point between the two hydrogens (*ρ*_BCP_ = 0.0111; ∇^2^*ρ*_BCP_ = 0.0469 a.u.). However, the short distance between the BCP and RCP (0.397 Bohr) puts in doubt the stability of this critical point.

Regarding X = Cl, despite the stabilization of the *ortho* derivatives mentioned above (Table 1), no *ortho* interactions, such as the halogen bonding [38], are observed. Precisely, no spatial requirements for its creation are fulfilled because the σ-hole of Cl is located in a different region than the lone pair of the N atom (Lewis base) is directed, as seen on the electrostatic potential (ESP) map (Figure S4c, where σ-hole appears as a reddish spot above the Cl atom). Therefore, the observed stabilization comes from the electronic interactions between Cl and the endo N atom. An interesting observation can be made from the plot *ρ*_BCP_ (CCl) versus *d*_CCl_, shown in Figure 5c. Two distinctive series of points are noticeable. One of them corresponds to the *ortho* systems, while the second to other systems (*meta*, *para*, quinolines substituted in all-carbon ring). It indicates the existence of some specific stabilizing electronic interactions occurring between the endo-N and the Cl atom in the *ortho* position. Their stabilizing character is confirmed by *E*_rel_ (negative values, Table 1); for the remaining derivatives, the obtained *E*_rel_ values are positive. Taking into account *ρ*_BCP_(CCl), its range of variability for *ortho* systems is more than twice as extensive as for other derivatives. Almost the same applies to changes in the length of the CCl bonds. The most substantial stabilizing electronic interactions are observed for Cl-triazine (highest electron density at BCP of CCl bond), followed by 2-Cl- and 4-Cl-pyrimidine. In the first case, the two *ortho* interactions are enhanced by the *para* endo-N effect. In 2-Cl-pyrimidine, there are two *ortho* interactions, while, in 4-Cl-pyrimidine, there are *ortho* and *para* interactions. Moreover, in all *ortho* systems, the obtained cSAR values reveal the ED ability of the Cl group (Figure 5d).

The only proximity interactions of Cl detected by RDG analysis are close Cl∙∙∙H contacts in 4- and 5-Cl-quinolines, classified as van der Waals interactions (Figure 6). Additionally, in 8-Cl-quinoline, Cl∙∙∙N contact is classified as partly steric and partly van der Waals in character.

The results of the RDG function analysis reveal interesting proximity effects of the NO_2_ group. As shown in Figure 6, the NO∙∙∙HC interaction is detectable in the RDG plots as an attractive interaction with strength in between the hydrogen bonding and the van der Waals interaction (sign[λ_2_(r)]**∙***ρ*(r) < 0). For NH∙∙∙N interaction, no hydrogen bond critical point is observed, but, instead, the O∙∙∙HC angle and O∙∙∙H distance are more favorable (in 2-NO_2_-pyridine, respectively, 95° and 2.396 Å) compared to the NH∙∙∙N ones. Although the two O∙∙∙H interactions are present in *meta* and *para* derivatives, the O∙∙∙H distance is 0.03 to 0.05 Å longer (Table S1). RDG plots, presented in Figure 6, show that they can be classified as van der Waals interactions. The geometry of the *ortho* NO_2_ group is deformed by the interaction with the endo-N atom, which strengthens the NO∙∙∙HC interaction. Interestingly, RDG isosurfaces suggest that NO∙∙∙N contacts are not purely repulsive (sign[λ_2_(r)]**∙***ρ*(r) > 0). A contribution of the van der Waals interaction can be seen as a green-colored fragment (corresponding to the sign[λ_2_(r)]**∙***ρ*(r) ~ 0) on the RDG isosurface between the O and N atoms (Figure 6). On the other hand, there is no indication of attractive interaction between these atoms on ESP plots (Figure S4a). Namely, the ESP value on the surface in the vicinity of the O and N atoms is negative.

## 4. Conclusions

The study aimed to investigate to what extent the endocyclic nitrogen atom(s) in mono-substituted heterocyclic derivatives affects the energetic, structural, and electronic parameters of the substituent. For this purpose, the following substituents were selected: nitro, chlorine, and amine, while pyridine, pyrimidine, pyrazine, 1,3,5-triazine, and quinoline were chosen as the studied systems.

Using the relative energy parameter, *E*_rel_, based on the energetic effect of the appropriate homodesmotic reaction with benzene as a reference system, allows for comparing the properties of the substituents. The values obtained for *para* substituted NH_2_-, Cl-, and NO_2_-pyridine are −3.02, 0.23, and 3.01 kcal/mol, respectively. Thus, it has been documented that the presence of the endo-N atom in the *para* position stabilizes the amino group and destabilizes the nitro group in relation to benzene derivatives. In general, stabilization/destabilization increases when the endo-N atoms are close to the substituent (*ortho*). For the amine and nitro groups, the greatest proximity effect is observed in their triazine derivatives (*E*_rel_ = −15.84 and 8.55 kcal/mol, respectively). Moreover, it has been shown that *E*_rel_ can be obtained from the additivity of interactions.

The use of the cSAR (charge of the substituent active region) model allows for comparison of changes in the local electronic structure, thus revealing the reverse substituent effect. The electron-donating/accepting strength of the considered substituents in heterocycles depends on the substitution position, mainly on the proximity effects. The electron-attracting power of NO_2_ and Cl groups decreases, while the electron-donating ability of the amino group increases, compared to that observed in the case of benzene derivatives. The determined ranges of variability for cSAR are 0.191, 0.186, and 0.159, respectively, for the NH_2_, NO_2_, and Cl groups.

Changes in the energy and electronic structure of the substituents are accompanied by geometric changes. The results of the analysis of bond lengths and valence angles showed many of their mutual correlations. In the case of nitro six-membered derivatives, changes in structural parameters generally follow the Bent-Walsh rule. Moreover, in the case of six-membered amine heterocycles, the substituent’s energetic, electronic, and geometric characteristics are highly interdependent.

Proximity interactions are the main reason for such significant changes in the energetic, electronic, and structural properties of the substituent. It is documented by changes in the lengths of CN, NO, and NH bonds, as well as the YNY and ∠CNY angles. The use of RDG (reduced density gradient) analysis has proven particularly useful in studying the nature of these non-covalent intramolecular interactions. In the case of the NO_2_ proximity effects, the obtained results indicate the attractive NO∙∙∙HC interaction with strength between the hydrogen bonding and the van der Waals interaction and suggest that the NO∙∙∙N contacts are not purely repulsive. In the case of the amino group, the NH∙∙∙N interaction in 8-NH_2_-quinoline is the only such interaction that can be unambiguously classified as a hydrogen bond both on the basis of the RDG analysis and the bond critical point criteria. Moreover, in 4- and 5-NH_2_-quinolines, close NH∙∙∙HC contacts are detected by RDG as van der Waals interactions. The only proximity Cl interactions, detected by the RDG analysis, are close Cl∙∙∙H contacts in 4- and 5-Cl-quinolines, classified as van der Waals interactions. However, in 8-Cl-quinoline, the Cl∙∙∙N contact is classified as partly steric and partly van der Waals in character.

In summary, the substituent properties significantly depend on the number of endo-nitrogen atoms in the ring, as well as their arrangement and the position of the substitution in relation to them.

## Figures and Tables

**Table 1:** *E*_rel_ values (in kcal/mol) obtained from Equation (2) and from the additivity of proximity effects. Int. columns depict types of interactions occurring for a given system: *o*—*ortho*, *m*—*meta*, *p*—*para*.

		X = NO_2_		X = NH_2_		X = Cl	
R-X	Int.	*E*_rel_	*E*_rel-add_	*E*_rel_	*E*_rel-add_	*E*_rel_	*E*_rel-add_
2-X-pyridine	(*o*)	2.27		−6.71		−1.50	
3-X-pyridine	(*m*)	2.63		0.04		1.01	
4-X-pyridine	(*p*)	3.01		−3.02		0.23	
2-X-pyrimidine	(*o, o*)	6.29	4.54	−11.99	−13.42	−0.51	−3.00
4-X-pyrimidine	(*o, p*)	4.97	5.28	−10.08	−9.73	−1.35	−1.27
5-X-pyrimidine	(*m, m*)	5.29	5.26	−0.05	0.08	1.99	2.02
X-pyrazine	(*o, m*)	5.02	4.90	−6.99	−6.67	−0.60	−0.49
X-triazine	(*o, o, p*)	8.55	7.55	−15.84	−16.44	−0.56	−2.77
2-X-quinoline	(*o*)	1.74	1.89	−7.61	−6.89	−2.25	−1.50
3-X-quinoline	(*m*)	1.95	2.25	0.03	−0.14	0.92	1.01
4-X-quinoline	(*p*)	6.10	7.55	−2.19	−2.06	0.67	0.23
5-X-quinoline		4.12	4.54	1.01	0.96	0.94	
6-X-quinoline		0.59	−0.38	−0.49	−0.18	0.39	
7-X-quinoline		1.13	−0.38	−1.08	−0.18	0.35	
8-X-quinoline		7.33	4.54	−3.38	0.96	2.60	

**Table 2:** Electronic properties of substituents in the studied heterocycles.

	X = NO_2_			X = NH_2_			X = Cl		
R-X	cSAR	∆pEDA	∆sEDA	cSAR	∆pEDA	∆sEDA	cSAR	∆pEDA	∆sEDA
2-X-pyridine	−0.078	−0.032	0.002	0.163	−0.053	0.065	0.016	−0.015	−0.002
3-X-pyridine	−0.128	−0.010	−0.024	0.103	−0.042	0.060	−0.030	−0.002	−0.017
4-X-pyridine	−0.097	−0.024	−0.019	0.140	−0.049	0.060	−0.004	−0.011	−0.012
2-X-pyrimidine	0.000	−0.166	0.096	0.244	−0.068	0.057	0.083	−0.031	−0.016
4-X-pyrimidine	−0.038	−0.042	−0.016	0.213	−0.064	−0.041	0.054	−0.027	−0.010
5-X-pyrimidine	−0.118	−0.013	−0.019	0.113	−0.042	−0.023	−0.020	−0.005	−0.029
X-pyrazine	−0.065	−0.035	−0.004	0.181	−0.056	0.068	0.031	−0.016	0.005
X-triazine	0.043	−0.207	0.114	0.292	−0.075	0.058	0.124	−0.039	0.000
2-X-quinoline	−0.079	−0.034	−0.016	0.172	−0.074	−0.014	0.017	−0.019	−0.009
3-X-quinoline	−0.143	−0.014	−0.017	0.103	−0.045	−0.011	−0.035	−0.003	−0.004
4-X-quinoline	−0.093	−0.102	0.063	0.143	−0.063	−0.013	0.008	−0.010	−0.008
5-X-quinoline	−0.127	−0.057	0.046	0.101	−0.054	0.077	−0.021	−0.003	−0.002
6-X-quinoline	−0.137	−0.013	−0.018	0.108	−0.054	0.074	−0.032	−0.003	−0.008
7-X-quinoline	−0.130	−0.017	−0.019	0.115	−0.048	0.063	−0.027	−0.005	−0.011
8-X-quinoline	−0.130	−0.195	0.128	0.104	−0.053	0.057	−0.028	−0.010	−0.020

**Table 3:** Geometric properties of substituents in NO_2_ and NH_2_ derivatives. Bond lengths are given in Å, angles in °.

	X = NO_2_					X = NH_2_				
R-X	*d*_CN_	∠ONO	∆*α*	*d*_NO(H)_	*d*_NO(N)_	*d*_CN_	∠HNH	∆*α*	*d*_NH(H)_	*d*_NH(N)_
2-X-pyridine	1.509	125.7	2.46	1.227	1.213	1.383	115.3	−1.14	1.007	1.009
3-X-pyridine	1.478	125.1	2.27	1.224	1.223	1.394	112.5	−1.40	1.009	1.009
4-X-pyridine	1.488	125.3	2.53	1.222	1.222	1.383	114.1	−1.63	1.008	1.008
2-X-pyrimidine	1.503	127.0	3.13	1.218	1.218	1.363	119.4	−0.75	1.006	1.006
4-X-pyrimidine	1.515	126.3	2.66	1.225	1.211	1.366	117.6	−1.32	1.006	1.008
5-X-pyrimidine	1.474	125.5	2.30	1.223	1.223	1.388	112.8	−1.37	1.009	1.009
X-pyrazine	1.504	126.1	2.27	1.226	1.213	1.377	115.7	−1.14	1.007	1.009
X-triazine	1.500	127.6	3.22	1.216	1.216	1.349	120.9	−0.90	1.006	1.006
2-X-quinoline	1.512	125.6	2.40	1.227	1.213	1.382	115.4	−1.01	1.008	1.009
3-X-quinoline	1.473	124.9	2.20	1.225	1.225	1.393	112.5	−1.25	1.009	1.009
4-X-quinoline	1.484	125.0	2.17	1.224	1.223	1.382	113.5	−1.73	1.007	1.008
5-X-quinoline	1.479	124.3	1.87	1.227	1.224	1.399	111.2	−1.52	1.010	1.010
6-X-quinoline	1.480	124.7	2.25	1.225	1.225	1.394	112.6	−1.32	1.009	1.009
7-X-quinoline	1.482	124.8	2.20	1.223	1.225	1.392	112.9	−1.40	1.009	1.009
8-X-quinoline	1.478	125.7	2.05	1.225	1.218	1.377	116.3	−1.87	1.007	1.010

**Table 4:** Geometric properties of substituents in Cl derivatives. Bond lengths are given in Å, angles in °.

	2-Cl-Pyridine	3-Cl-Pyridine	4-Cl-Pyridine	2-Cl-Pyrimidine	4-Cl-Pyrimidine	5-Cl-Pyrimidine	Cl-Pyrazine	Cl-Triazine	2-Cl-Quinoline	3-Cl-Quinoline	4-Cl-Quinoline	5-Cl-Quinoline	6-Cl-Quinoline	7-Cl-Quinoline	8-Cl-Quinoline
*d*_CCl_	1.763	1.754	1.751	1.75	1.752	1.746	1.754	1.741	1.767	1.754	1.755	1.762	1.758	1.758	1.748
∆*α*	1.26	1.28	1.2	1.09	1.03	1.18	1.05	0.97	1.34	1.38	1.17	1.22	1.41	1.33	0.73

**Table 5:** Correlation coefficients, *cc*, between pairs of parameters, obtained for six-membered heterocycles. Data for X = NO_2_ (red) and X = NH_2_ (blue) are presented.

	*E*_rel_		*d*_CN_		∠YNY		∆*α*		*α*		cSAR		*d*_NY(H)_		*d*_NY(N)_		∆pEDA		∆sEDA	
*E*_rel_			0.19		0.89		0.74		0.55		0.82		−0.79		−0.22		−0.83		0.79	
	
*d*_CN_	0.97				0.55		0.41		0.73		0.62		0.09		−0.95		−0.34		0.31	
	
∠YNY	−0.99		−0.98				0.89		0.86		0.98		−0.72		−0.49		−0.93		0.91	
	
∆*α*	−0.78		−0.70		0.79				0.83		0.92		−0.86		−0.22		−0.95		0.92	
	
*α*	−0.91		−0.81		0.90		0.90				0.90		−0.51		−0.60		−0.84		0.85	
	
cSAR	−0.99		−0.99		0.99		0.78		0.88				−0.70		−0.53		−0.93		0.89	
	
*d*_NY(H)_	0.97		0.94		−0.96		−0.67		−0.87		−0.95				−0.23		0.87		−0.85	
	
*d*_NY(N)_	0.83		0.89		−0.89		−0.66		−0.74		−0.88		0.78				0.22		−0.19	
	
∆pEDA	0.99		0.98		−0.99		−0.76		−0.89		−0.99		0.97		0.87				−0.99	
	
∆sEDA	−0.11		0.00		0.08		0.28		0.33		0.09		0.00		−0.14		−0.08			
	

**Table 6:** Correlation coefficients, *cc*, between pairs of parameters, obtained for six-membered Cl-heterocycles.

*E*_rel_		*d*_CCl_		∆*α*		*α*		cSAR		∆pEDA		∆sEDA			
		−0.41		0.38		−0.75		−0.58		0.63		−0.76		*E*_rel_	
	
				0.58		−0.02		−0.45		0.41		0.24		*d*_CCl_	
	
						−0.55		−0.84		0.83		−0.43		∆*α*	
	
								0.87		−0.86		0.55		α	
	
										−0.99		0.49		cSAR	
	
												−0.47		∆pEDA	
	
														∆sEDA	
	
								
	

## Data Availability

The data presented in this study are available in the article and in the associated Supplementary Materials.

## Supplementary Materials

The following are available online, Figure S1: Relations between electron density at NO(H) bond critical point (BCP) and its length, electron density at CN BCP and its length, electron density at NO(N) BCP and its length, electron density at CN BCP and cSAR(X) of substituent for the nitro derivatives of studied heterocycles; for the amino derivatives relation between electron density at CN BCP and its length, and the cSAR(X). Figure S2: Relationships between electronic characteristics of the substituents, cSAR(X) and α angle for X = NH_2_, NO_2_, and Cl. Figure S3: RDG isosurfaces (at RDG = 0.50). Figure S4: Electrostatic potential mapped onto the *ρ* = 0.01 a.u. isosurface for 2-NO_2_-pyridine, 2-NH_2_-pyridine, and 2-Cl-pyridine. Table S1: Electron densities at CX, NY(H), and NY(N) bond critical points, and Y∙∙∙N(H), Y∙∙∙H(N) distances (Y = O in NO_2_, H in NH_2_); Table S2: Data regarding the *E*_rel_, *d*_CX_, α, ∠YNY, ∆α, cSAR(X), *d*_NY(H)_, *d*_NY(N)_, ∆pEDA(X), ∆sEDA(X), ∠CNO(N), ∠CNO(H), and rotation of the nitro group in all studied heterocycles, as well as in benzene and naphthalene NO_2_, NH_2_, and Cl derivatives.

## Sample Availability

Samples of the compounds are not available from the authors.
