# Supplementary material for: Energetic and Geometric Characteristics of the Substituents: Part 2: The Case of NO2, Cl, and NH2 Groups in Their Mono-Substituted Derivatives of Simple Nitrogen Heterocycles
Source: Molecules. 2021 Oct 29;26(21):6543. doi: 10.3390/molecules26216543 (PMC8588088; doi:10.3390/molecules26216543)
Supplement: Supplementary file 1 [file molecules-26-06543-s001.zip › molecules-1418383-supplementary.pdf]

Supplementary Materials (SM)

Energetic and Geometric Characteristics of the Substituents: Part 2: The Case of NO_2, Cl, and NH_2 Groups in Their Mono-Substituted Derivatives of Simple Nitrogen Heterocycles

Paweł A. Wieczorkiewicz[1,*], Halina Szatylowicz[1], Tadeusz M. Krygowski[2,*]

1 Faculty of Chemistry, Warsaw University of Technology, Noakowskiego 3, 00-664 Warsaw, Poland
2 Department of Chemistry, University of Warsaw, Pasteura 1, 02-093 Warsaw, Poland
* Correspondence: tmkryg@chem.uw.edu.pl (T.M.K.); pwieczorkiewicz@outlook.com (P.A.W.)

Table of Contents

Figure S1. Relations between electron density at NO(H) bond critical point (BCP) and its length, electron density at CN BCP and its length, electron density at NO(N) BCP and its length, electron density at CN BCP and cSAR(X) of substituent for the nitro derivatives of studied heterocycles; for the amino derivatives relation between electron density at CN BCP and its length, and the cSAR(X). — SM 2

Figure S2. Relationships between electronic characteristics of the substituents, cSAR(X) and α angle for X = NH_2, NO_2, and Cl. — SM 3

Figure S3. RDG isosurfaces (at RDG = 0.50) — SM 3

Figure S4. Electrostatic potential mapped onto the ρ = 0.01 a.u. isosurface for 2-NO_2-pyridine, 2-NH_2-pyridine and 2-Cl-pyridine. — SM 6

Table S1. Electron densities at CX, NY(H) and NY(N) bond critical points, and Y···N(H), Y···H(N) distances (Y = O in NO2, H in NH_2). — SM 8

Table S2. Data regarding the E_{rel}, d_{CX}, α, ∠YNY, $\Delta\alpha$, cSAR(X), $d_{NY(H)}$, $d_{NY(N)}$, ΔpEDA(X), ΔsEDA(X), ∠CNO(N), ∠CNO(H) and rotation of the nitro group in all studied heterocycles as well as in benzene and naphthalene NO_2, NH_2 and Cl derivatives. — SM 10

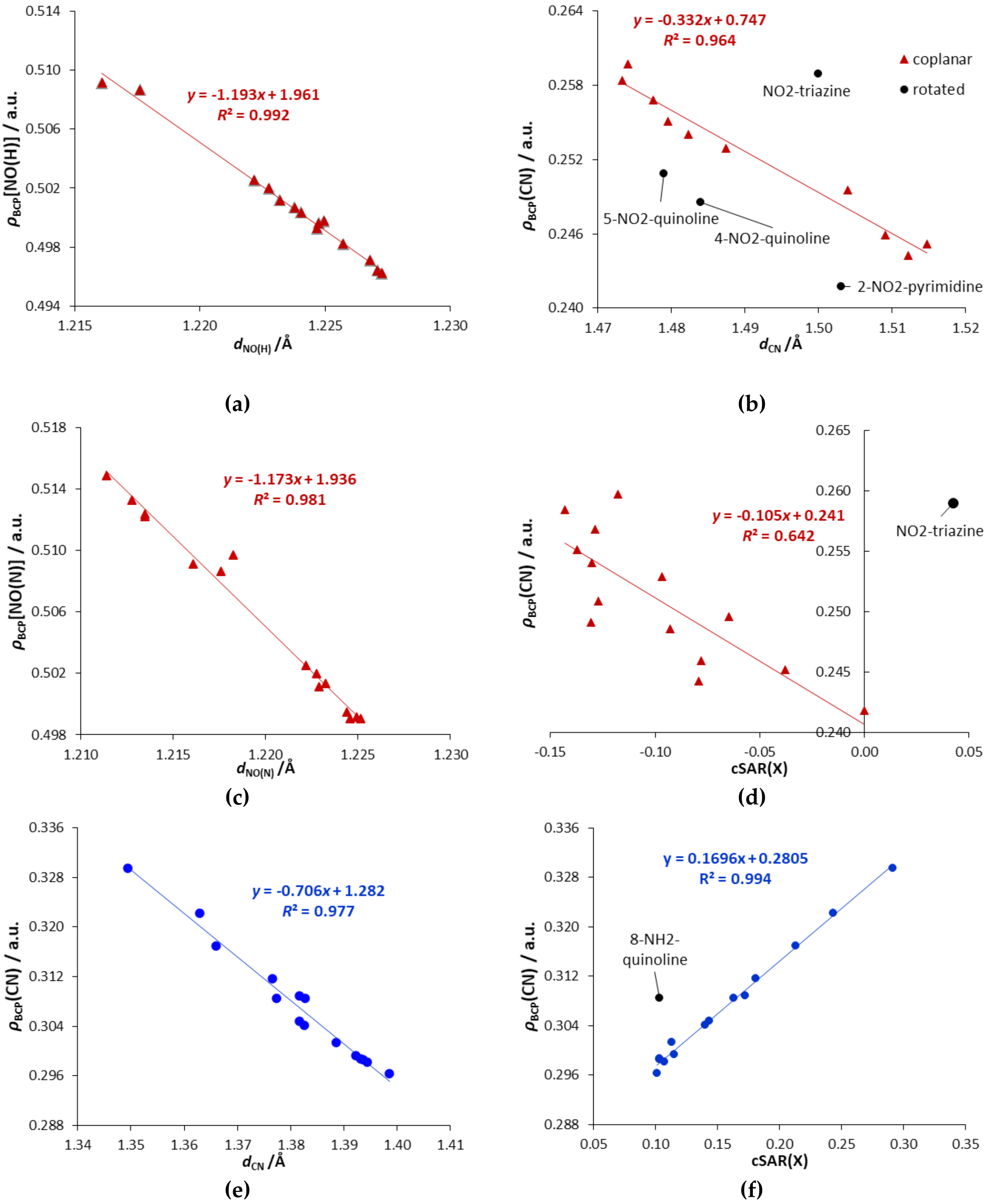

Figure S1. Relations between electron density at NO(H) bond critical point (BCP) and its length (a), electron density at CN BCP and its length (b), electron density at NO(N) BCP and its length (c), electron density at CN BCP and cSAR(X) of substituent (d) for the nitro derivatives of studied heterocycles; for the amino derivatives relation between electron density at CN BCP and its length (e), and the cSAR(X) (f).

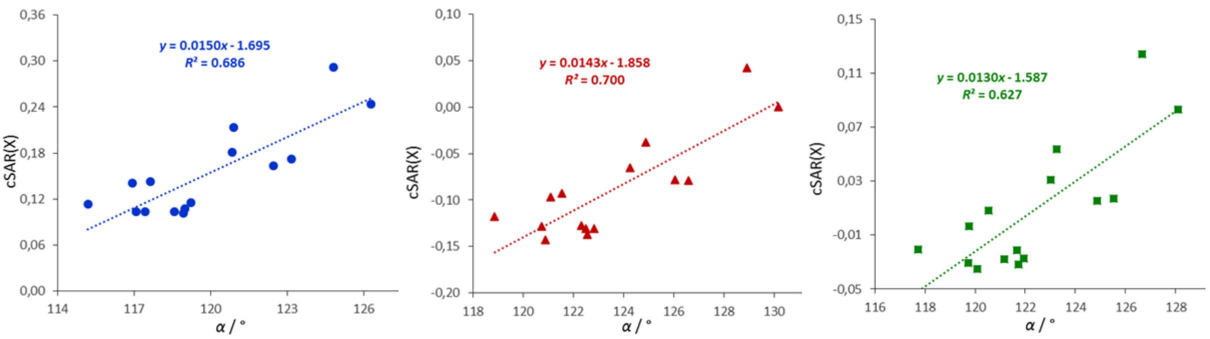

(a) (b) (c)

Figure S2. Relationships between electronic characteristics of the substituents, cSAR(X) and α angle for X = NH_2 (a), NO_2 (b), and Cl (c); for nitro derivatives only planar systems are considered.

Figure S3. RDG isosurfaces (at RDG = 0.50). Isosurfaces are colored according to the value of sign[λ_2(r)]·ρ(r); highly negative values in blue (indicating strong H-bonding interaction), around zero in green (van der Waals interaction), highly positive in red (steric repulsion).

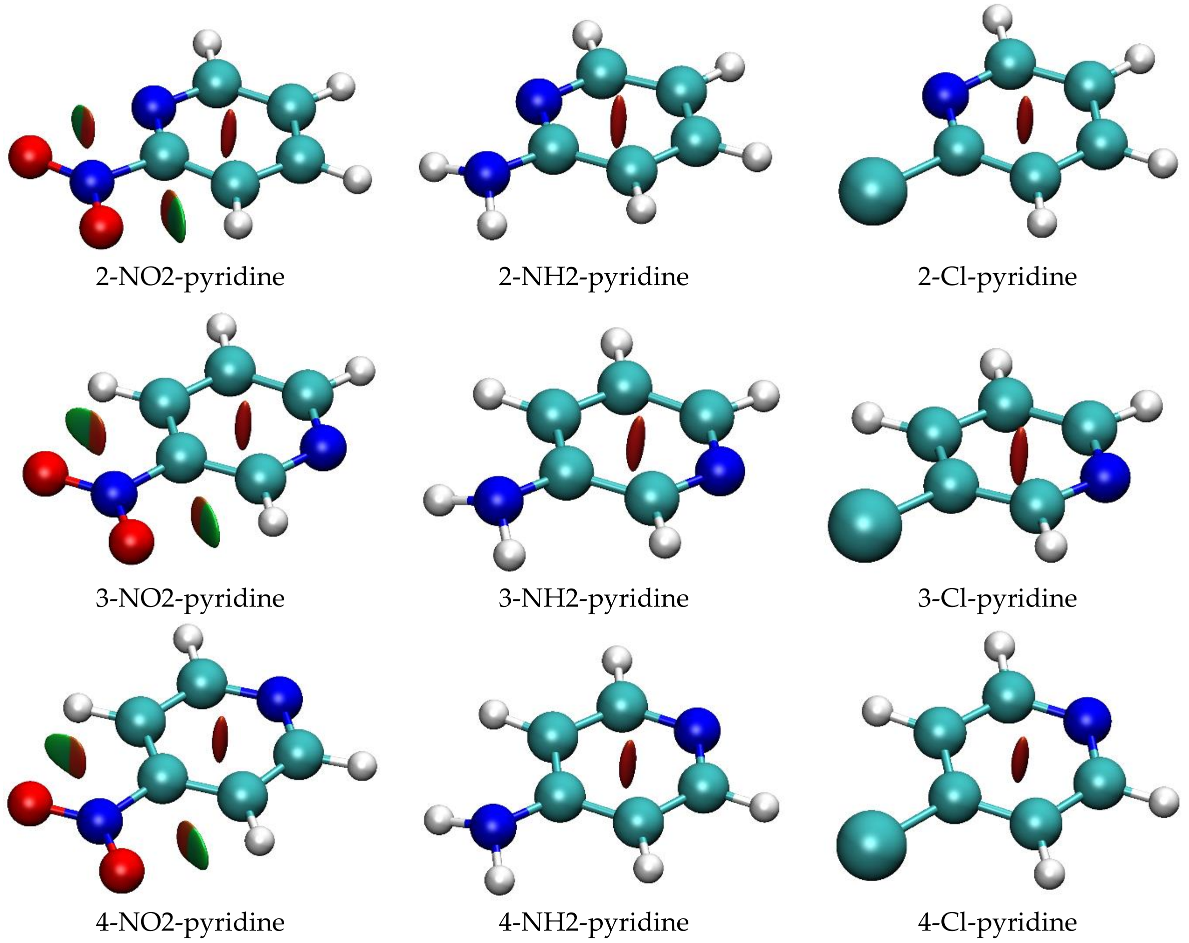

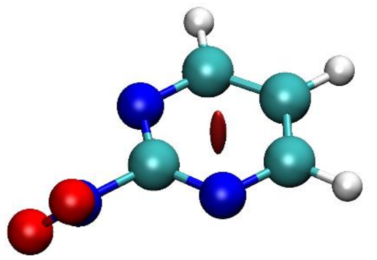
2-NO2-pyrimidine

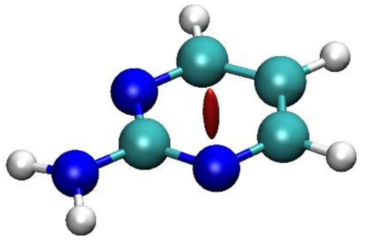
2-NH2-pyrimidine

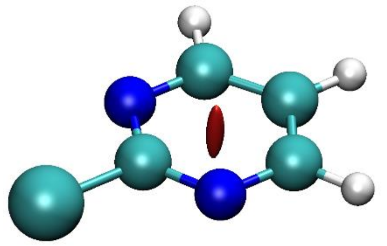
2-Cl-pyrimidine

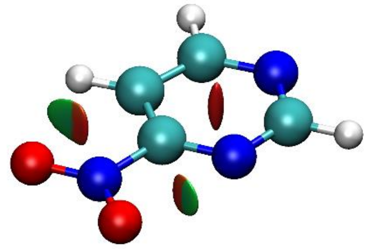
4-NO2-pyrimidine

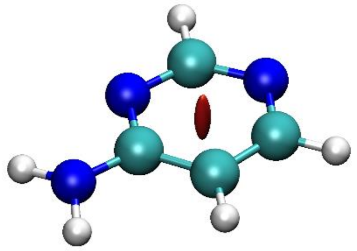
4-NH2-pyrimidine

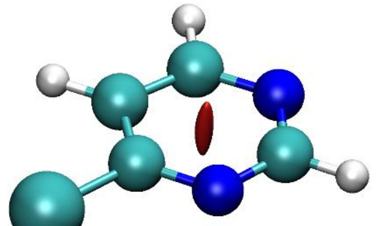
4-Cl-pyrimidine

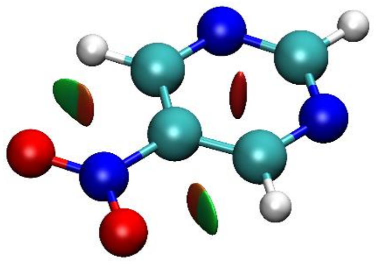
5-NO2-pyrimidine

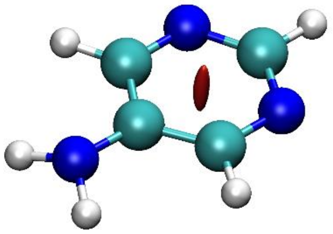
5-NH2-pyrimidine

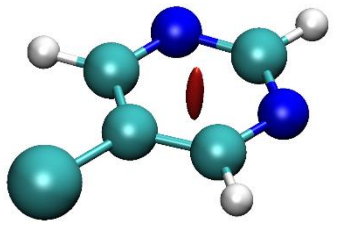
5-Cl-pyrimidine

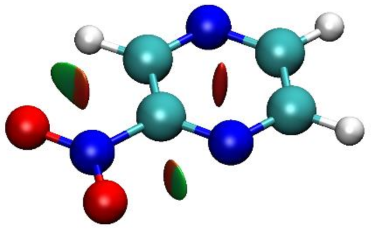
NO2-pyrazine

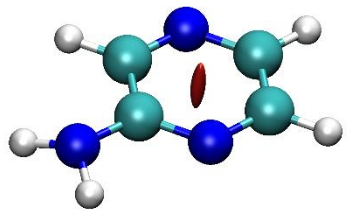
NH2-pyrazine

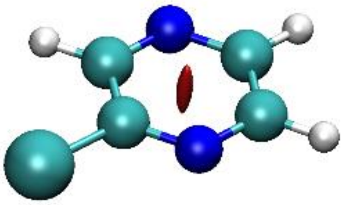
Cl-pyrazine

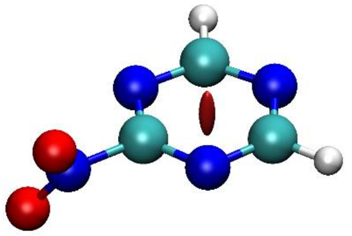
NO2-triazine

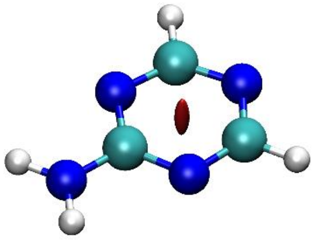
NH2-triazine

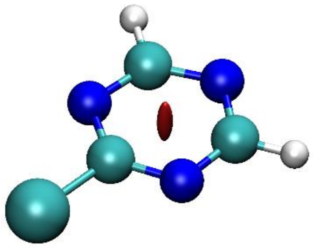
Cl-triazine

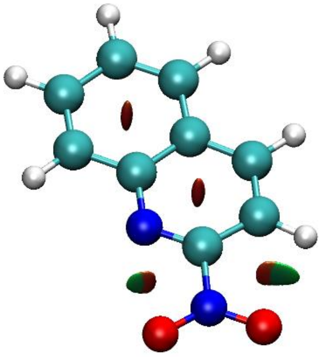

2-NO2-quinoline

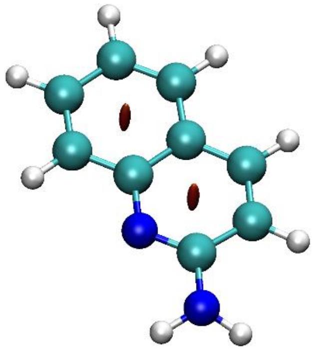

2-NH2-quinoline

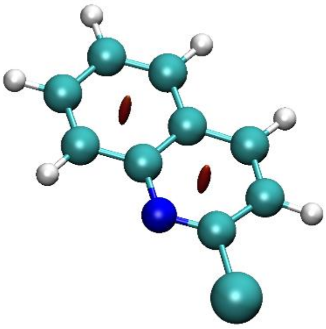

2-Cl-quinoline

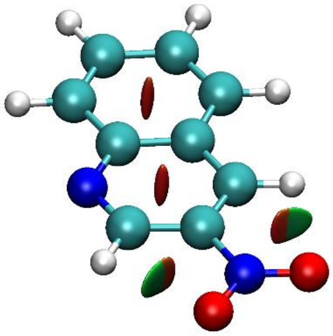

3-NO2-quinoline

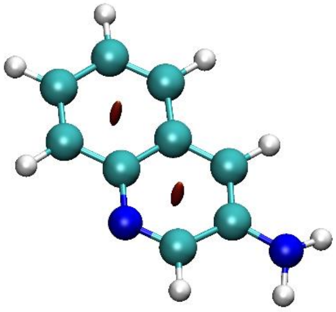

3-NH2-quinoline

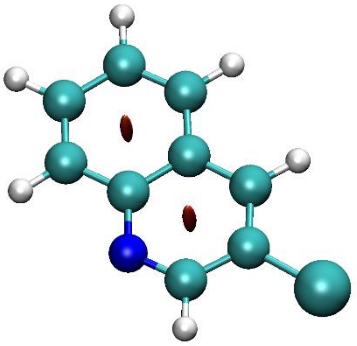

3-Cl-quinoline

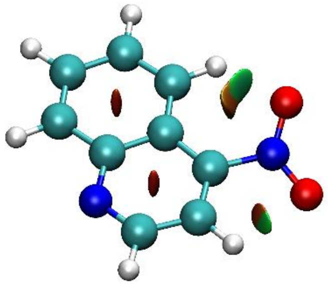

4-NO2-quinoline

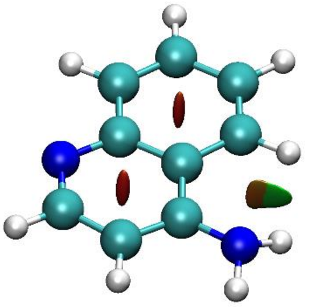

4-NH2-quinoline

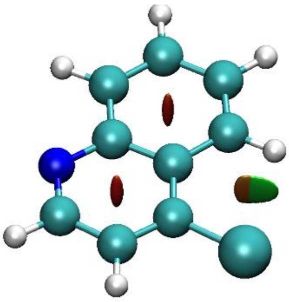

4-Cl-quinoline

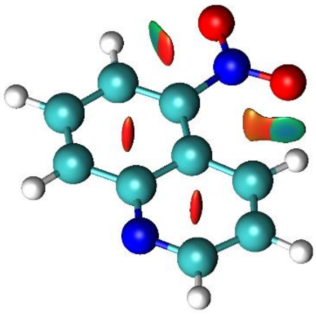

5-NO2-quinoline

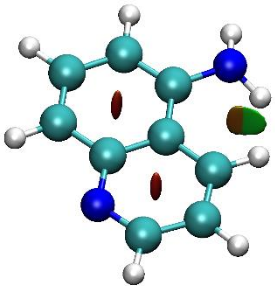

5-NH2-quinoline

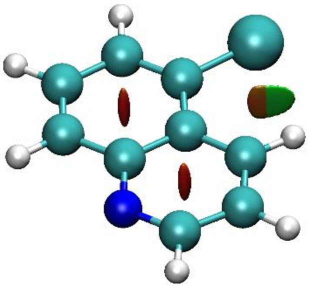

5-Cl-quinoline

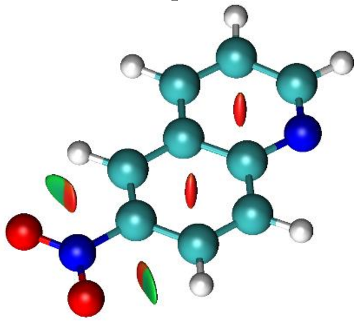

6-NO2-quinoline

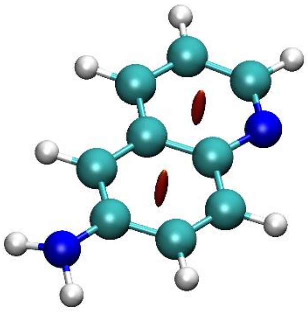

6-NH2-quinoline

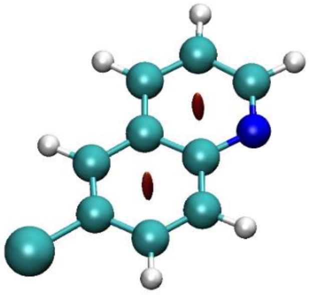

6-Cl-quinoline

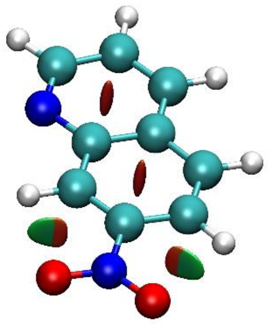

7-NO2-quinoline

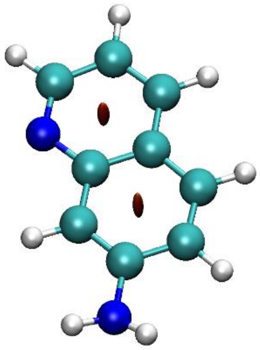

7-NH2-quinoline

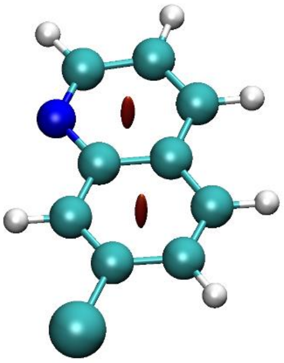

7-Cl-quinoline

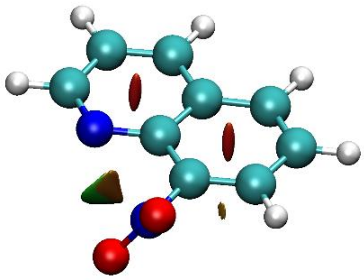

8-NO2-quinoline

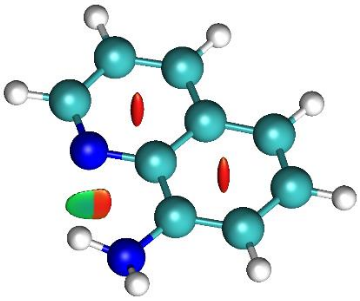

8-NH2-quinoline

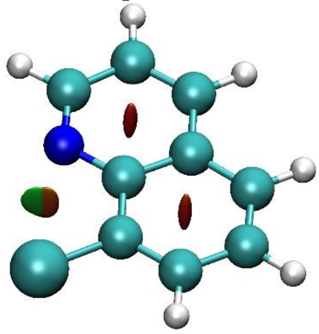

8-Cl-quinoline

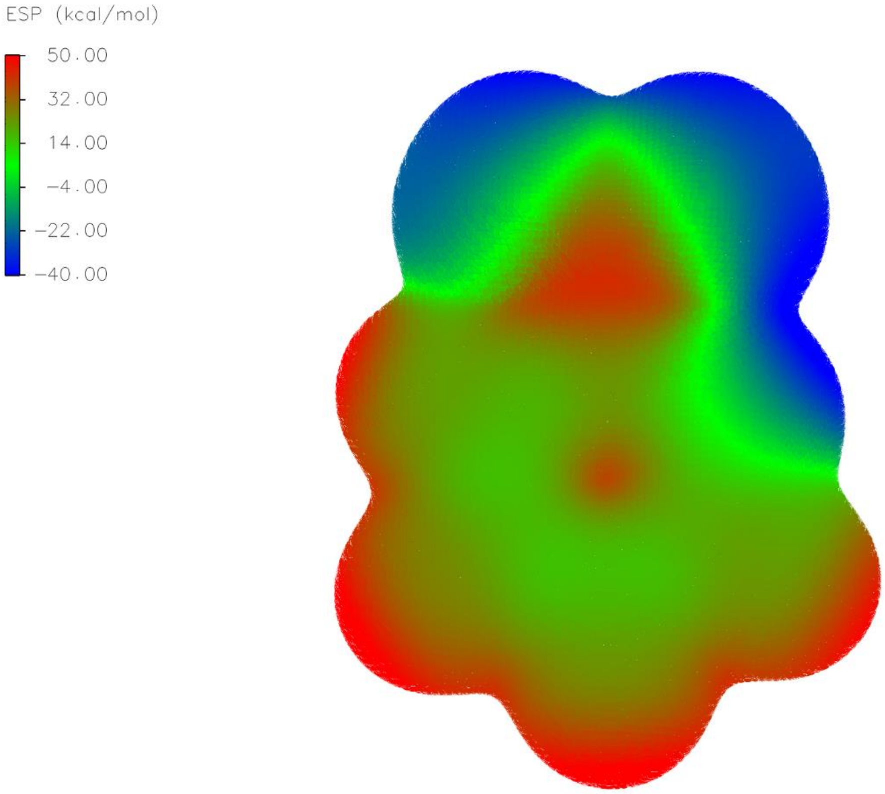

(a)

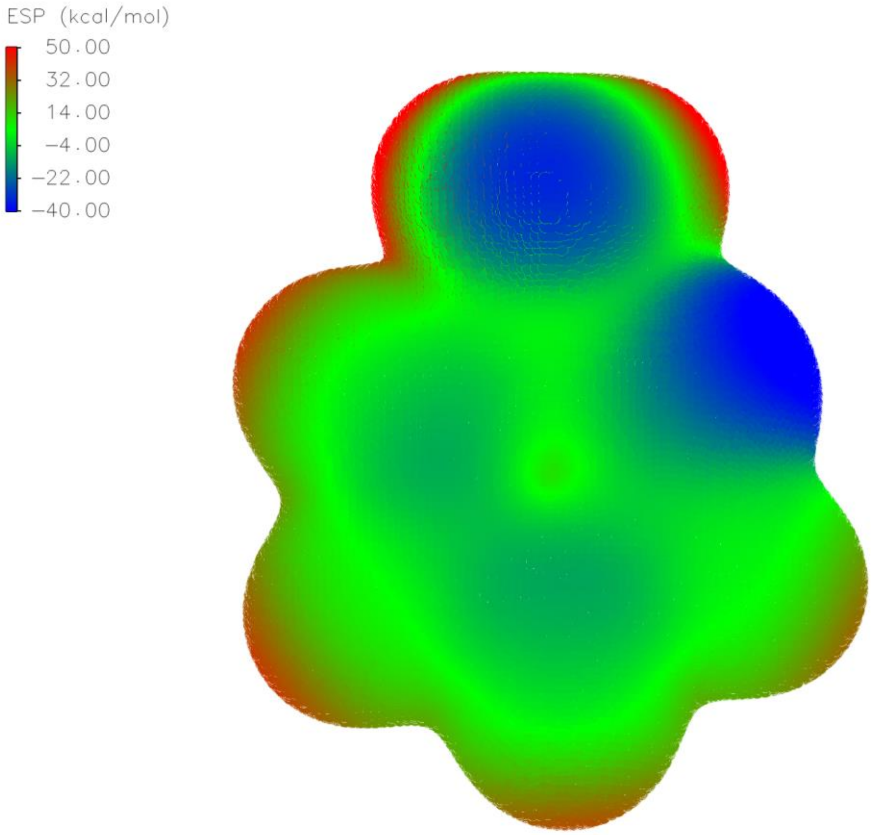

(b)

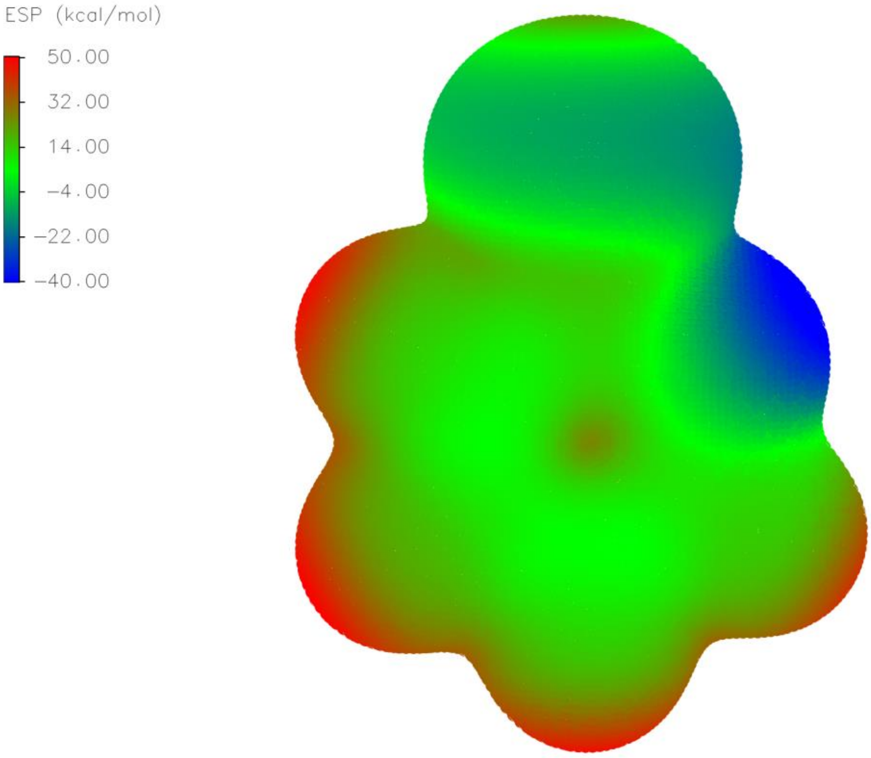

(c)

Figure S4. Electrostatic potential mapped onto the ρ = 0.01 a.u. isosurface for 2-NO_2-pyridine (a), 2-NH_2-pyridine (b) and 2-Cl-pyridine (c).

Table S1. Electron densities at CX, NY(H) and NY(N) bond critical points, and Y···N(H), Y···H(N) distances (Y = O in NO2, H in NH_2). $d_{Y\cdots N(H)}$ denotes the distance between Y (Y = O in NO_2 and H in NH_2) and *ortho* N atom. In asymmetric non-*ortho* derivatives it indicated distance to the H atom of the *ortho* C-H group closer to the *endo* N. $d_{Y\cdots H(N)}$ denotes the distance to the *ortho* C-H group further to the *endo* N, or, in the case of double *ortho* derivatives, second *endo* N atom.

	ρ_{BCP}(CX) / a.u.	ρ_{BCP}[NY(N)] / a.u.	ρ_{BCP}[NY(H)] / a.u.	$d_{Y\cdots N(H)}$ / Å	$d_{Y\cdots H(N)}$ / Å
**2-NO_2-pyridine**	0.2459	0.5123	0.4964	2.653	2.396
**3-NO_2-pyridine**	0.2568	0.5004	0.5013	2.435	2.425
**4-NO_2-pyridine**	0.2529	0.5025	0.5025	2.442	2.442
**2-NO_2-pyrimidine**	0.2418	0.5087	0.5087	2.821	2.821
**4-NO_2-pyrimidine**	0.2452	0.5149	0.4993	2.662	2.439
**5-NO_2-pyrimidine**	0.2597	0.5020	0.5020	2.467	2.467
**NO_2-pyrazine**	0.2496	0.5132	0.4982	2.662	2.437
**NO_2-triazine**	0.2589	0.5091	0.5091	2.887	2.887
**2-NO_2-quinoline**	0.2442	0.5122	0.4962	2.649	2.390
**3-NO_2-quinoline**	0.2584	0.4991	0.4996	2.425	2.419
**4-NO_2-quinoline**	0.2485	0.5012	0.5007	2.478	2.268
**5-NO_2-quinoline**	0.2508	0.4995	0.4971	2.210	2.390
**6-NO_2-quinoline**	0.2551	0.4991	0.4998	2.398	2.391
**7-NO_2-quinoline**	0.2540	0.4990	0.5012	2.422	2.389
**8-NO_2-quinoline**	0.2491	0.5097	0.4925	2.940	2.726
**2-NH_2-pyridine**	0.3085	0.3389	0.3404	2.430	2.499
**3-NH_2-pyridine**	0.2986	0.3395	0.3395	2.428	2.487
**4-NH_2-pyridine**	0.3042	0.3402	0.3402	2.474	2.474
**2-NH_2-pyrimidine**	0.3223	0.3405	0.3405	2.475	2.475
**4-NH_2-pyrimidine**	0.3169	0.3389	0.3409	2.452	2.541
**5-NH_2-pyrimidine**	0.3014	0.3392	0.3392	2.494	2.494
**NH_2-pyrazine**	0.3116	0.3384	0.3402	2.460	2.510
**NH_2-triazine**	0.3295	0.3401	0.3401	2.491	2.491
**2-NH_2-quinoline**	0.3088	0.3388	0.3403	2.441	2.461
**3-NH_2-quinoline**	0.2987	0.3393	0.3395	2.399	2.499
**4-NH_2-quinoline**	0.3048	0.3403	0.3410	2.416	2.056
**5-NH_2-quinoline**	0.2963	0.3399	0.3396	2.120	2.356
**6-NH_2-quinoline**	0.2981	0.3400	0.3398	2.403	2.445
**7-NH_2-quinoline**	0.2993	0.3400	0.3399	2.457	2.410
**8-NH_2-quinoline**	0.3085	0.3380	0.3404	2.286	2.511

**pyridine2Cl**	0.1923
**pyridine3Cl**	0.1938
**pyridine4Cl**	0.1952
**pyrimidine2Cl**	0.2003
**pyrimidine4Cl**	0.1973
**pyrimidine5Cl**	0.1968
**pyrazineCl**	0.1960
**triazineCl**	0.2047
**quinoline2Cl**	0.1909
**quinoline3Cl**	0.1934
**quinoline4Cl**	0.1934
**quinoline5Cl**	0.1901
**quinoline6Cl**	0.1920
**quinoline7Cl**	0.1924
**quinoline8Cl**	0.1964

Table S2. Data regarding the E_{rel} (kcal/mol), d_{CX} (Å), α (°), ∠YNY (°), $\Delta\alpha$ (°), cSAR(X), $d_{NY(H)}$ (Å), $d_{NY(N)}$ (Å), ΔpEDA(X), ΔsEDA(X), ∠CNO(N) (°), ∠CNO(H) (°),and rotation of the nitro group (°) in all studied heterocycles as well as in benzene and naphthalene NO_2, NH_2 and Cl derivatives. Naming of geometry parameters is explained in Figure 4 in the manuscript.

RX	E_{rel}	d_{CX}	α	∠YNY	$\Delta\alpha$	cSAR(X)	$d_{NY(H)}$	$d_{NY(N)}$	ΔpEDA(X)	ΔsEDA(X)	∠CNY(N)	∠CNY(H)	NO_2 rotation
benzNO_2	0	1.4812	122.3	124.7	2.32	-0.138	1.225	1.225	0	0	117.7	117.7	0
pyridine2NO_2	2.27	1.5092	126.1	125.7	2.46	-0.078	1.227	1.213	-0.032	0.002	118.2	116.1	0
pyridine3NO_2	2.63	1.4776	120.7	125.1	2.27	-0.128	1.224	1.223	-0.010	-0.024	117.5	117.4	0
pyridine4NO_2	3.01	1.4875	121.1	125.3	2.53	-0.097	1.222	1.222	-0.024	-0.019	117.4	117.4	0
pyrimidine2NO_2	6.29	1.5031	130.1	127.0	3.13	0.000	1.218	1.218	-0.166	0.096	116.5	116.5	56
pyrimidine4NO_2	4.97	1.5148	124.9	126.3	2.66	-0.038	1.225	1.211	-0.042	-0.016	117.9	115.8	0
pyrimidine5NO_2	5.30	1.4742	118.9	125.5	2.30	-0.118	1.223	1.223	-0.013	-0.019	117.2	117.2	0
pyrazineNO_2	5.01	1.5041	124.3	126.1	2.27	-0.065	1.226	1.213	-0.035	-0.004	118.1	115.8	0
triazineNO_2	8.55	1.5001	128.9	127.6	3.22	0.043	1.216	1.216	-0.207	0.114	115.5	116.2	66
naphtalene1NO_2	4.54	1.4870	122.1	123.3	1.20	-0.131	1.226*	1.226	-	-	117.7	119.0	0
naphtalene2NO_2	-0.38	1.4781	122.5	124.6	2.22	-0.148	1.226*	1.225	0.009	0.000	117.5*	118.0	0
quinoline2NO_2	1.75	1.5123	126.6	125.6	2.40	-0.079	1.227	1.213	-0.034	-0.016	118.3	116.1	0
quinoline3NO_2	1.95	1.4733	120.9	124.9	2.20	-0.143	1.225	1.225	-0.014	-0.017	117.4	117.7	0
quinoline4NO_2	6.10	1.4841	121.5	125.0	2.17	-0.093	1.224	1.223	-0.102	0.063	117.9	117.1	35

quinoline5NO$_2$	4.12	1.4791	122.3	124.3	1.87	-0.127	1.227	1.224	-0.057	0.046	118.2	117.5	28
quinoline6NO$_2$	0.59	1.4796	122.6	124.7	2.26	-0.137	1.225	1.225	-0.013	-0.018	117.4	117.9	0
quinoline7NO$_2$	1.13	1.4824	122.8	124.8	2.20	-0.130	1.223	1.225	-0.017	-0.019	117.9	117.3	0
quinoline8NO$_2$	7.34	1.4782	122.5	125.7	2.05	-0.130	1.225	1.218	-0.195	0.128	117.4	116.8	58
benzNH$_2$	0	1.3983	118.5	112.1	-1.42	0.094	1.009	1.009	0	0	115.6	115.6	
pyridine2NH$_2$	-6.71	1.3827	122.5	122.5	-1.14	0.163	1.007	1.009	-0.053	0.065	114.7	117.9	
pyridine3NH$_2$	0.04	1.3940	117.1	117.1	-1.40	0.104	1.009	1.009	-0.042	0.060	115.9	116.1	
pyridine4NH$_2$	-3.02	1.3826	116.9	116.9	-1.63	0.140	1.008	1.008	-0.049	0.060	117.4	117.4	
pyrimidine2NH$_2$	-11.99	1.3629	126.3	119.4	-0.75	0.244	1.006	1.006	-0.068	0.057	118.1	118.1	
pyrimidine4NH$_2$	-10.08	1.3660	120.9	117.6	-1.32	0.213	1.006	1.008	-0.064	-0.041	116.8	120.0	
pyrimidine5NH$_2$	-0.05	1.3880	115.2	112.8	-1.37	0.114	1.009	1.009	-0.042	-0.023	116.5	116.5	
pyrazineNH$_2$	-6.99	1.3766	120.8	115.7	-1.14	0.181	1.007	1.009	-0.056	0.068	115.5	118.4	
triazineNH$_2$	-15.84	1.3494	124.8	120.9	-0.90	0.292	1.006	1.006	-0.075	0.058	119.5	119.5	
naphtalene1NH$_2$	0.96	1.3984	119.32	111.4	-1.55	0.097	1.010*	1.009	-	-	114.4*	115.9	
naphtalene2NH$_2$	-0.18	1.3972	118.99	112.2	-1.30	0.097	1.009	1.009	-0.005	-0.002	115.7*	115.7	
quinoline2NH$_2$	-7.61	1.3816	123.2	115.4	-1.01	0.172	1.008	1.009	-0.074	-0.014	114.9	118.1	

quinoline3NH_2	0.03	1.3930	117.4	112.5	-1.25	0.104	1.009	1.009	-0.045	-0.011	116.0	116.2
quinoline4NH_2	-2.19	1.3817	117.6	113.5	-1.73	0.143	1.007	1.008	-0.063	-0.013	116.4	117.9
quinoline5NH_2	1.01	1.3985	118.9	111.3	-1.52	0.101	1.010	1.010	-0.054	0.077	114.4	116.0
quinoline6NH_2	-0.49	1.3944	119.0	112.6	-1.32	0.108	1.009	1.009	-0.054	0.074	116.0	116.1
quinoline7NH_2	-1.08	1.3922	119.2	112.9	-1.40	0.115	1.009	1.009	-0.048	0.063	116.2	116.4
quinoline8NH_2	-3.38	1.3774	118.6	116.3	-1.87	0.104	1.007	1.010	-0.053	0.057	114.9	118.0
benzCl	0	1.7604			1.42	-0.051			0	0		
pyridine2Cl	-1.50	1.7635	124.9		1.26	0.016			-0.015	-0.002	116.7	118.5
pyridine3Cl	1.01	1.7540	119.7		1.28	-0.030			-0.002	-0.017	119.8	120.5
pyridine4Cl	0.23	1.7513	119.8		1.21	-0.004			-0.011	-0.012	120.1	120.1
pyrimidine2Cl	-0.51	1.7499	128.1		1.09	0.083			-0.031	-0.016	116.0	116.0
pyrimidine4Cl	-1.35	1.7524	123.2		1.03	0.054			-0.027	-0.010	117.2	119.6
pyrimidine5Cl	1.99	1.7460	117.7		1.18	-0.020			-0.005	-0.029	121.1	121.1
pyrazineCl	-0.60	1.7545	123.0		1.05	0.031			-0.016	0.005		
triazineCl	-0.56	1.7407	126.7		0.97	0.124			-0.039	0.000		
naphtalene1Cl	0.85	1.7623	122.1		1.23	-0.038			-	-	118.2*	119.7

**naphtalene2Cl**	0.01	1.7599	121.8	0.89	-0.052	-	-	118.4*	119.8
**quinoline2Cl**	-2.25	1.7666	125.5	1.34	0.017	-0.019	-0.009	117.2	117.3
**quinoline3Cl**	0.92	1.7540	120.1	1.38	-0.035	-0.003	-0.004	118.8	121.1
**quinoline4Cl**	0.67	1.7547	120.5	1.166	0.008	-0.010	-0.008		
**quinoline5Cl**	0.94	1.7616	121.7	1.224	-0.021	-0.003	-0.002		
**quinoline6Cl**	0.39	1.7580	121.7	1.410	-0.032	-0.003	-0.008		
**quinoline7Cl**	0.35	1.7575	122.0	1.328	-0.027	-0.005	-0.011		
**quinoline8Cl**	2.60	1.7478	121.2	0.730	-0.028	-0.010	-0.020	119.5	119.3

*bond or angle in naphthalene derivative towards the substituted six-membered ring or in the direction where the second six-membered ring is further from the substituted ring.
